# Percutaneous Treatment of Left Main Disease: A Review of Current Status

**DOI:** 10.3390/jcm12154972

**Published:** 2023-07-28

**Authors:** Dario Pellegrini, Alfonso Ielasi, Maurizio Tespili, Giulio Guagliumi, Giuseppe De Luca

**Affiliations:** 1 Division of Cardiology, IRCCS Ospedale Galeazzi-Sant’Ambrogio, Via Cristina Belgioioso, 173, 20161 Milan, Italy; dar.pellegrini@gmail.com (D.P.);; 2 Division of Cardiology, AOU “Policlinico G. Martino”, Via Consolare Valeria, 1, 98124 Messina, Italy; 3 Department of Clinical and Experimental Medicine, University of Messina, Piazza Pugliatti, 1, 98122 Messina, Italy

**Keywords:** left main disease, stent, PCI, intracoronary imaging

## Abstract

Percutaneous treatment of the left main coronary artery is one of the most challenging scenarios in interventional cardiology, due to the large portion of myocardium at risk the technical complexity of treating a complex bifurcation with large branches. Our aim is to provide un updated overview of the current indications for percutaneous treatment of the left main, the different techniques and the rationale underlying the choice for provisional versus upfront two-stent strategies, intravascular imaging and physiology guidance in the management of left main disease, and the role of mechanical support devices in complex high-risk PCI.

## 1. Introduction

Coronary artery disease still represents the leading cause of mortality [1], with the outcome still unsatisfactory in high-risk subsets of patients [2,3]. Large interests are focused on the identification of new risk factors [4,5] and improvement in technologies [6,7,8,9] to allow the percutaneous treatment of the vast majority of patients. A crucial population is currently represented by those patients with significant unprotected left main (ULM) disease. Percutaneous treatment of the left main coronary artery is one of the most challenging scenarios in interventional cardiology due to the large portion of myocardium at risk and to the technical difficulty of treating a complex bifurcation with large branches. Therefore, we aimed to provide un updated overview of the current indications for percutaneous treatment of the left main, the different techniques, the rationale underlying the choice for provisional versus upfront two-stent strategies, and the use of intravascular imaging and physiology guidance in the management of left main disease.

## 2. Anatomy and Relevant Aspects during Treatment

Disease of the ULM is a frequent finding during coronary angiography, and occurs in up to 4–9% of cases [10,11].

The left main stem of the left coronary artery (LCA) delivers blood to 60% of myocardium in the case of a dominant right coronary artery (RCA), or even to >90% of the heart in the case of a dominant left coronary artery (LCA). Considering its crucial function, the threshold for angiographic significance of a stenosis was set at 50%, compared to 70% of all other districts (except for proximal left anterior descending) because early observations reported a relevant survival after CABG in patients with a stenosis between 50 and 70% [12,13].

Disease can involve the proximal (ostial) segment of the LM, the mid segment (i.e., the shaft), or the distal LM at the level of the bifurcation with the left anterior descending (LAD) and the left circumflex (LCX) arteries. Still, the disease is rarely focal, and despite the angiographic evidence of a limited disease, it is often more extensive and involves also the distal branches, with an involvement of the bifurcation in up to 80% of cases [14,15,16]. Atherosclerosis usually affects the lateral walls of the bifurcation, with a relative sparing of the flow divider. This could be linked to a different distribution of the endothelial shear stress, with areas of low shear stress being more prone to development and progression of atherosclerotic plaques, while higher shear stress (such as at the carina) seems to be protective [17,18,19].

## 3. Available Evidence

Four major randomized controlled trials (RCTs) compared CABG and PCI for ULM disease: the SYNTAX (2009) [20], PRECOMBAT (2011) [21], EXCEL (2016) [15], and NOBLE (2020) [14,22] (Table 1). In these trials, ULM stenosis was defined as a stenosis >50%, except for EXCEL, which had a higher cutoff of 70% stenosis, or a positive fractional flow reserve (FFR) in case of a lower degree of angiographic severity. Moreover, EXCEL and NOBLE recommended intravascular ultrasound (IVUS) guidance in case of PCI.

In SYNTAX trial, all-cause death at 10-year follow-up was similar for PCI with the first generation paclitaxel-eluting stent (Taxus, Boston Scientific) and CABG (27% vs. 28%, respectively) [24]. The rate of major adverse cardiac and cerebrovascular events (MACCE, available up to a 5-year follow-up) showed a similar trend (15.8% vs. 13.7% [*p* 0.44] for PCI and CABG, respectively, at 1 year [20] and 36.9% vs. 31.0% [*p* 0.12] at 5 years [25]), with a higher rate of strokes in the CABG group and repeat revascularization in the PCI group. Subgroup analyses uncovered different performance of PCI according to the degree anatomy complexity of the disease, with good outcomes for low-to-medium complexity and worse outcomes for high SYNTAX scores compared to CABG [26,27].

The PRECOMBAT study confirmed the findings, with a non-significant difference in a composite endpoint of MACCE, but a higher rate of TLR in PCI (with first generation sirolimus-eluting stent, Cypher [Cordis, Johnson & Johnson, Miami Lakes, FL, USA]) compared to CABG, which persisted at an extended 10-year follow-up [21,28]. The EXCEL trial [15] enrolled only patients with low-to-mid SYNTAX scores, and confirmed the data, with a 30-day superiority of PCI with an everolimus-eluting stent (Xience, Abbott Vascular Inc., Santa Clara, CA, USA) due to a lower rate of periprocedural myocardial infarctions (MI), but a late catch-up, mainly due to increased TLR, which led to a similar rate of MACCE [29]. Finally, the NOBLE trial [14] (88% use of the second generation Biomatrix biolimus-eluting stent-Biosensor/Jiwei Co., Shandong, China) detected a significant reduction in MACCE in the CABG group compared to PCI, mainly due to a reduction in TLR, strokes, and MI. Still, it should be acknowledged that per protocol the trial did not adjudicate periprocedural MI, and there was a higher rate of stent thrombosis compared to the EXCEL trial (5% in the NOBLE vs. 1.8% in the EXCEL, at 5 years [22,29]) and the usual high rate of late strokes (5% in the PCI group vs. 2% in the CABG group at 5 years, compared to 2.9% for PCI and 3.7% for CABG in the EXCEL at 5 years).

A meta-analysis of these trials demonstrated no significant differences in the global rate of deaths between the two strategies (PCI vs. CABG: HR 1.10, 95% CI 0.91–1.32). The higher rate of repeat revascularization with PCI observed in the individual trials was confirmed (HR 1.78 compared to CABG, 95% CI 1.51–2.10), along with a higher rate of myocardial infarctions (HR 1.34 compared to CABG, 95% CI 1.08–1.67) [30].

## 4. Current Guideline Recommendations for Unprotected Left Main PCI

The most updated guidelines on ULM revascularization are the 2018 European Society of Cardiology (ESC)/European Association of Cardio-Thoracic Surgery (EACTS) Guidelines on Myocardial Revascularization [31] and the 2021 American college of Cardiology (ACC)/Society for Cardiovascular Angiography (SCAI) Guidelines on Coronary Artery Revascularization [32]. The 2018 ESC/EACTS guidelines provide a strong recommendation for coronary artery bypass graft (CABG) in ULM disease across the entire spectrum of disease complexity, thanks to the robust evidence available in the literature (class of recommendation I, level of evidence LoE A). On the other hand, PCI is currently not recommended in complex disease (i.e., SYNTAX [Synergy Between PCI with Taxus and Cardiac Surgery] [20] score ≥ 33; class III, LoE B), as most of the studies comparing PCI and CABG excluded this group of patients. In case of lower complexity, PCI is considered a viable alternative, with a class I LoE A recommendation in case of low complexity (SYNTAX score ≤ 22) and a class IIa LoE A recommendation in case of intermediate complexity (i.e., SYNTAX score between 23 and 32) [31]. The 2021 ACC/SCAI guidelines share the strong recommendation for CABG (class I LoE B), while PCI is recommended when results are expected to be comparable to CABG and disease is of low-to-medium anatomic complexity (classe IIa, LoE B-non randomized) [32].

Obviously, the long-term benefits of CABG may come at the cost of a higher rate of acute adverse events, especially in high-risk, elderly, or frail patients, which may benefit from the lower invasiveness of PCI. Predicted surgical risk plays an important role in decision making. Both the European System for Cardiac Operative Risk Evaluation (EuroSCORE II) (www.euroscore.org/calc.html, last accessed on 23 July 2023) and the Society of Thoracic Surgeons (STS) score (http://riskcalc.sts.org, last accessed on 23 July 2023) are available to evaluate procedural risk. However, as stated in the 2018 ESC/EACTS guidelines [31], no score can provide a perfect prediction of reality, mainly due to limited external validation, relevance of some variables not considered by current scores (such as frailty) and limited applicability in specific contexts. Thus, individualized decisions should be made in specific conditions based on clinical judgment.

## 5. Provisional Technique: Rationale and Technical Aspects

The debate around the best stenting strategy for ULM (and bifurcations overall) has been ongoing for a long time. The DEFINITION trial proposed two criteria to define the complexity of a bifurcation, i.e., (for distal LM) a SB stenosis ≥70% and a length of disease ≥10 mm. In case the ULM lesion featured one of the two criteria, it was deemed as complex. In the trial, only 30% of the patients had complex bifurcations, and although suffering from a higher rate of adverse events compared to those with simple lesions, they had better outcomes with a two-stent strategy compared to a one-stent technique. Still, 70% of patients had non-complex lesions, and benefitted from a one-stent strategy [33].

This approach was embraced by the consensus documents of the European Bifurcation Club, which identified a predicted difficult re-access to the SB and a significant disease of the SB with a lesion length ≥5 mm [34,35] as criteria for an upfront two-stent strategy. The suggested treatment for all other cases (the majority of lesions) would be provisional strategy.

The philosophy of this strategy is to implant a stent on the main vessel–main branch (MV-MB) axis, optimize the result with balloons, and avoid the unnecessary deployment of an additional stent in the side branch (SB) in case of good result, which is associated per se with a higher risk of target lesion failure (TLF) and target lesion revascularization (TLR). Thus, it is important to highlight that provisional strategy does not preclude the final deployment of two stents, but it tries to avoid their routine use and rely on a second stent only if strictly necessary.

The technical steps for a provisional stenting technique are described in detail elsewhere [36], and exceed the purpose of this paper. Still, it is noteworthy to highlight some key points.

Usually, the LAD will be considered the MB of the bifurcation due to the larger diameter and the larger portion of myocardium at potential jeopardy compared to LCX. Still, in selected cases of large LCX (e.g., in case of left dominance), an “inverted” provisional technique may be chosen, with a single stent along the MV-SB axis, especially in bifurcations with an angle ≥90° and a predicted easy re-cross of the stent struts.

The careful optimization of the implanted stent is of crucial importance, through the so-called “proximal optimization technique” (POT). This step should be considered mandatory, and requires the correct position of a properly sized balloon (sized 1:1 to the LM) at the level of the carina. POT allows for achieving multiple goals: the optimal degree of stent expansion, the complete apposition to the vessel wall, and the removal of stent struts from the ostium of the SB, which appose to the wall opposite to the flow divider and help achieve a good scaffolding of the SB. Moreover, POT is intended to facilitate rewiring of the SB through the most distal stent strut in the bifurcation and avoid abluminal rewiring, which can often occur and remain unnoticed. Dilatation of the SB ostium (either with kissing balloon inflation or with alternate inflations in the MB and SB, the so-called POT-side-POT technique) is not mandatory in provisional technique, but it is advisable in the setting of the ULM to facilitate future interventions, if needed. Nevertheless, repeat POT (re-POT) should always be the last step to restore the fractal geometry of the stent. In case of a suboptimal result in the SB (i.e., residual stenosis ≥70%, major dissection, or flow impairment), a second stent can be implanted in the SB. T-stent, T-and-protrusion (TAP), reverse culotte, or reverse crush are feasible strategies to complete the procedure and allow a seamless transition to a two-stent technique.

## 6. Two-Sent Technique: Rationale and Technical Aspects

Although the provisional strategy is recommended in most bifurcation lesions, those deemed as complex bifurcations involving both the MB and the SB were shown to have better long-term outcomes with the adoption of an upfront two-stent technique [36].

As stated in the previous section, the DEFINITION trial showed that complex bifurcation lesions, despite a global higher rate of adverse events compared to non-complex lesions, benefit from an upfront two-stent strategy [21]. This was confirmed in the DEFINITION II trial [37], which demonstrated the superiority of a two-stent strategy, with the clear superiority of a double-kissing crush (DK-crush) technique compared to culotte. In the DK-crush V trial, DK-crush showed superiority compared to the provisional strategy in the treatment of LM bifurcation lesions [38]. However, the EBC-MAIN study [39] in 482 patients proposed a stepwise provisional stent technique also in cases with involvement of both the MB and SB, with a second stent to be implanted only in case of impaired blood flow, residual SB stenosis ≥90%, threatened SB closure, or a dissection grade higher than type A. The vast majority of patients (78%) in the provisional group received only one stent, with a relevant reduction in procedural time. No differences were found in the rate of adverse events compared to an upfront two-stent strategy. Still, the authors recognized that the complexity of the treated lesions was lower compared to the DK-crush V trial, with a mean SYNTAX score of 23 compared to 31 and a SB lesion length of 7 mm compared to 16 mm of the DK-crush V. This difference was clear also in the rate of the implantation of the second stent in the provisional arms of the two trials, with a 22% rate in EBC-MAIN compared to a 45% rate in the DK-crush V.

Thus, despite some discrepancies in study results, complex lesions should benefit from an upfront two-stent strategy. The two major techniques are double-kissing crush (DK-crush) and culotte, with a slight advantage in the recommendations in the 2018 ESC guidelines for DK-crush (IIb B recommendation in case of upfront need for a two-stent strategy) [31].

The procedural steps of these techniques are described elsewhere [40], and exceed the purpose of this review. Compared to standard crush, the addition of two kissing balloon inflations during DK-crush was shown to allow better stent expansion, especially at the level of the SB, despite an increase in technical complexity. Similar to DK-crush, some operators tried to improve traditional culotte by adding additional steps of kissing balloon inflations and developed DK-culotte, which is supposed to benefit from similar improvements in procedural results and clinical outcomes as DK-crush. At the moment, bench tests favor the new technique [41], but there are no clinical data available.

## 7. Technical Considerations during PCI

Optimal visualization of the entire LM and quantification of lesion severity can be challenging [42], and requires special care from the operator. Left anterior oblique caudal view (the so-called “spider view”) is considered the traditional projection with which to approach bifurcation lesions and to assess the correct position of stents at the ostium of the LAD and LCX in order to avoid geographical miss or excessive protrusion, especially in two-stent techniques. However, a recent study based on computerized tomography (CT) [43] showed that a straight caudal view would be affected by the lowest degree of vessel foreshortening, and thus it would offer the optimal view of the bifurcation.

The ostium of the LM is best visualized in a left cranial view, as it delineates the border between the LM and the sinus of Valsalva. So, in case of a stent covering the entire length of the bifurcation, the operator would likely need to assess the lesion from at least two projections, especially when choosing the correct stent length. Nevertheless, operators should keep in mind that these indications were derived from mean values of a limited population [43], and case-by-case adjustments may be needed.

The operators should also consider the specifics of the available devices. In particular, the maximum expansion limit of stents can be a key element when planning the procedure, as the stent (sized according to the diameter of the distal vessel) needs to expand to the size of the LM. Stent platforms show significant variations in terms of range of postdilation, so not all of the available devices in the laboratory may adapt to a specific setting. An additional point to address is the expansion of the struts when performing dilation of the SB. Stents with more connectors are more resistant to longitudinal deformation, but at the same time have smaller cells and limited overexpansion, such as SYNERGY [Boston Scientific, Natick, MA, USA] Resolute Onyx [Medtronic Santa Rosa, CA, USA], and Orsiro [Biotronik, Buelach, Switzerland]. This issue may be more evident in the case of two-stent techniques such as TAP and culotte with a large SB, where stent in the SB may be constrained by the limited expansion of the cell of the MB stent (leading to the so-called “napkin ring sign”) and be prone to a higher risk of TLF [40,44].

## 8. Intracoronary Imaging Guidance before, during and after PCI

Ideally, an integration of intracoronary physiology and imaging data would be ideal to obtain a comprehensive assessment of the LM lesion. However, in a real-world setting, financial costs related to the equipment and time constraints limit the applicability of this strategy, and the operator needs to choose between the two.

Currently, intracoronary imaging has a class IIa recommendation in guiding LM treatment [19]. Moreover, in recent large randomized trials (although not specific for the LM setting) [45,46,47], intracoronary imaging was shown to improve long-term clinical outcomes, besides just the immediate procedural result. Currently, a dedicated trial is ongoing (the OPTIMAL, OPtimizaTIon of Left MAin PCI With IntravascuLar Ultrasound) [48], with the aim to assess the superiority of an IVUS-guided approach, compared to angiography-guided PCI, in the specific setting of LM, both for provisional and two-stent techniques. Thus, it is likely that the recommendation for its usage to guide complex PCI (including LM) will become even stronger in future consensus documents. IVUS is usually preferred to optical coherence tomography (OCT), thanks to its ability to spare dye and to be independent from blood clearance, a well-known limitation of OCT in the evaluation of ostial lesions. The superior resolution of OCT can provide relevant advantages, especially when evaluating post-procedural results, and allows for an easier detection of stent malapposition. Still, at the moment, IVUS has an explicit recommendation in guidelines [30,31] based on available literature.

Considering the advantages of intracoronary imaging, in a recent paper, the American College of Cardiology stated that intravascular imaging should become a routine part of PCI, especially in complex interventions [49].

**Figure 1:**
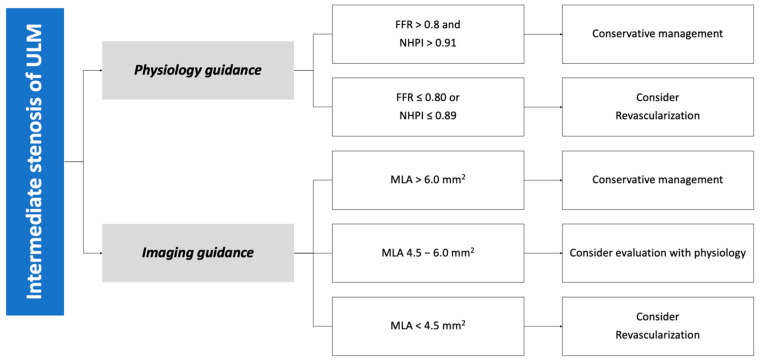
Algorithm for decision making in case of intermediate lesion of the left main stem. FFR: fractional flow reserve. NHPI: non-hyperemic physiology indices. MLA: minimum lumen area. ULM: unprotected left main.

According to IVUS studies, a minimum lumen area (MLA) > 6.0 mm^2^ in Western populations has good correlations with a FFR > 0.80 [50,51]. In prospective studies, the LITRO study [51] found that, over two years of follow-up, deferring LM lesions with a MLA > 6.0 mm^2^ was safe and provided similar outcomes compared to those patients undergoing revascularization. Furthermore, the few patients with a MLA < 6.0 mm^2^ not undergoing revascularization had a significant increase in adverse events. Due to the proximal position of the LM in the vasculature, its size does not seem to be influenced by body surface area or sex. Still, some variation was observed across the population [52], as in the Asian population, a cut-off of 4.5 mm^2^ was found to correlate with a FFR of 0.75 [53]. Thus, considering the available data, lesions with a MLA < 4.5 mm^2^ should be considered severe and deserve treatment. Treatment deferral and conservative management of any lesion with a MLA > 6.0 mm^2^ should be safe, while lesions with an MLA between 4.5 and 6.0 mm^2^ should be considered as a “grey zone” and assessed with additional tests, such as intracoronary physiology (Figure 1).

**Figure 2:**
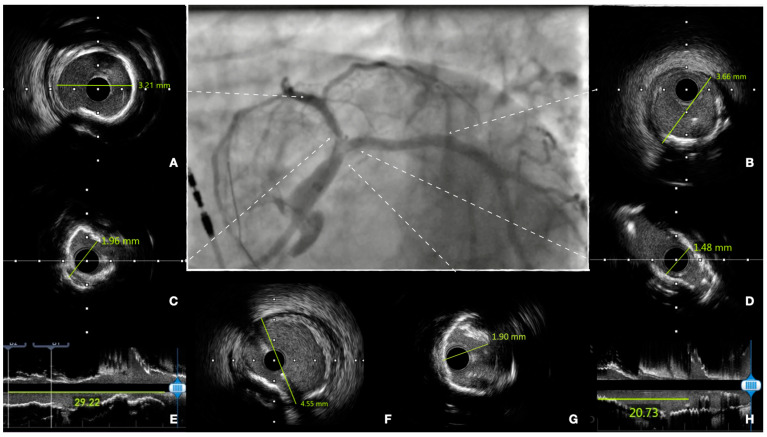
Pre-procedural assessment of left main (LM) disease. Center panel: coronary angiography, showing severe disease of the distal LM, which involves the origin of both the left anterior descending (LAD) and the left circumflex (LCX). Intravascular ultrasound assessment allows accurate measurement of the reference vessel diameter of the LAD (panel (**A**)), of the LCX (**B**), and of the LM (**F**), minimum lumen diameter of the LAD (**C**), of the LCX (**D**) and of the distal LM (**G**), and longitudinal length of the lesion in both vessels ((**E**,**H**) for LAD and LCX, respectively). Assessment of plaque morphology shows severe calcifications of both vessels, with an almost complete circumferential calcification at the level of the bifurcation. When possible, sizing is performed according to external elastic lamina when visible (e.g., (**B**)) or according to lumen (**A**), if plaque does not allow a clear visualization of the media of the vessel.

**Figure 3:**
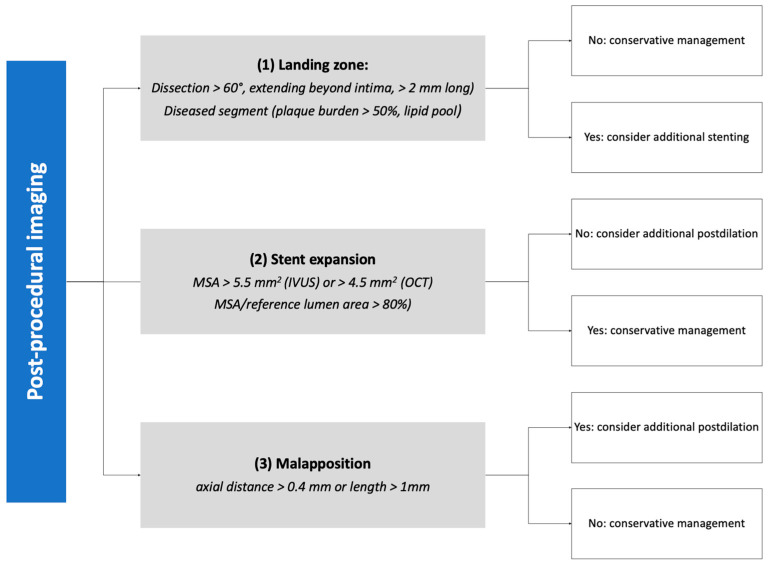
Algorithm for stent optimization based on post-procedural imaging. IVUS: intravascular ultrasound. MSA: minimum stent area. OCT: optical coherence tomography.

**Figure 4:**
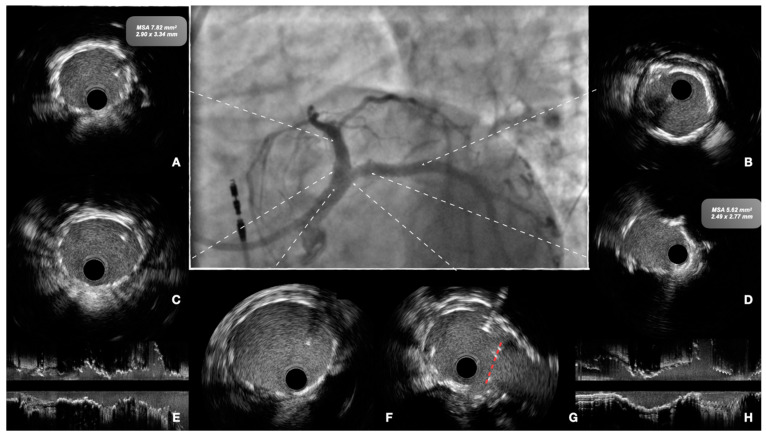
Final procedural assessment of the case presented in Figure 1. The patient was treated with lithotripsy and implantation of two stents, with a T-and protrusion (TAP) technique. Center panel: final angiographic view. Intravascular ultrasound shows good expansion and apposition of the stents and no residual dissection at the level of the distal landing of the left anterior descending (LAD) and left circumflex (LCX) arteries (panel (**A**,**B**), respectively) at the level of the ostia (**C**,**D**) and at the level of the proximal and distal left main (**F**,**G**). Neutral position of the neo-carina is shown in panel (**G**) (red dotted line). Longitudinal view of the stents in the LAD and LCX are shown in panels (**E**,**H**).

**Figure 5:**
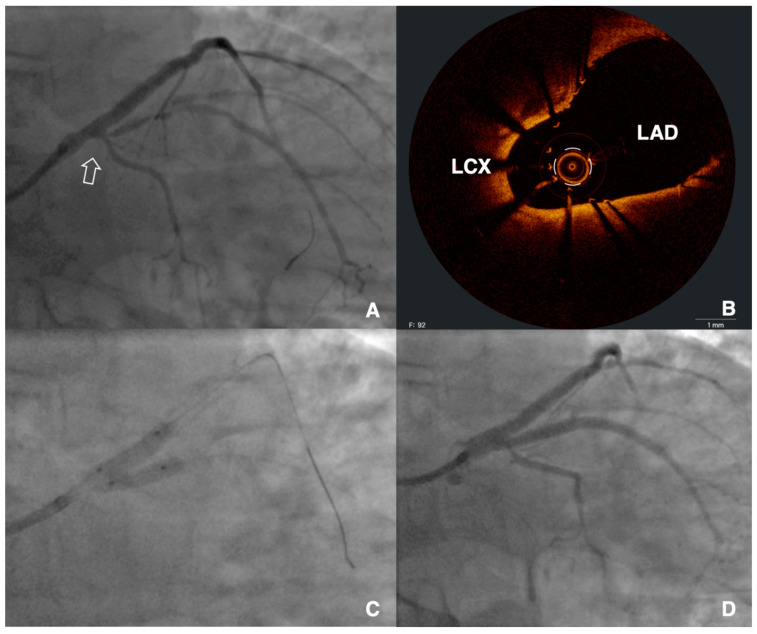
Intravascular detection of unintentional stent protrusion in the left main. The patient had a previous intervention in the left anterior descending (LAD), with stents trying to nail the ostium of the vessel. A staged procedure of the left circumflex (LCX) was planned due to severe stenosis up to the ostium (panel (**A**), arrow). Due to difficult delivery of devices in the LCX, optical coherence tomography was performed (**B**), showing unintentional protrusion of the “ostial” stent from the LAD in the left main, with good expansion but extensive malapposition and intraluminal passage of the LCX wire. The lesion was then treated with kissing balloon inflation (**C**) and implantation of a new stent from the LCX to the LM to complete a culotte technique. After final kissing balloon inflation, a good result was achieved in both vessels (**D**). MSA: minimum stent area.

When planning treatment, imaging can show vessel size (reference vessel diameter [RVD] of LM, LAD, and LCX), lesion length, plaque composition, and landing zones. Before stenting, imaging can show successful or inadequate lesion preparation; moreover, accurate measurement of vessel RVD can guide precise stent selection (Figure 2). At the end of the procedure, imaging can help assess expansion and apposition of the stent(s), the presence of geographical miss, edge dissection, and neutrality of the carina (Figure 3). This latter aspect is of particular relevance, as it is a common finding after POT due to too distal placement of the balloon. Conventional angiography cannot detect it, and it can lead to future TLF. Intracoronary imaging can easily detect carina shift, and allow for quick and effective correction (Figure 4). Unintentional protrusion of stent struts in the LM may be detected too, especially in the case of the previous implantation of stents trying to “nail the ostium” of the LAD or the LCX. In this case, intracoronary imaging may show the location and size of the stent, relationships with the MB and SB, and position of the wires to guide subsequent procedural steps and avoid unintentional crushing of previously implanted stents (Figure 5).

## 9. Coronary Physiology Guidance

Physiology guidance is a key element of modern state-of-the-art PCI. Both resting and hyperemic indexes proved to offer a reliable guidance for lesion treatment or deferral. Still, although several trials with thousands of patients demonstrated the beneficial impact of FFR and instantaneous wave-free ratio (iFR, the most studied non-hyperaemic index), unprotected LM was often excluded from such trials [54,55]. Thus, only few data exist on the use of coronary physiology to guide management and treatment. The largest study published so far involved 213 patients evaluated with FFR. At 5-year follow-up, patients with a negative FFR (i.e., >0.80) had similar outcomes compared to those undergoing surgical revascularization (CABG) due to FFR ≤ 0.80 [56]. At the moment, no relevant study exists on iFR, but it is reasonable to consider it a reliable tool for other non-LM lesions. More data from randomized trials are needed to consolidate the role of intracoronary physiology in LM management, but the available literature from non-LM trials is still sufficient to provide good evidence of the usefulness of these tools.

Considering the innate differences in the two groups of indexes, FFR may have an advantage over resting indexes due to the large territory of myocardium perfused by the LM, which may lead to a response to hyperemia, and a higher sensitivity of hyperemic indexes such FFR. Indeed, very proximal segments were associated more frequently with a mismatch between indexes, with a positive FFR and a false-negative iwFR [57].

When assessing lesion significance, the operator should perform measurements both in the LAD and LCX, considering the fractal geometry of the bifurcation. In case of involvement of the LM ostium, careful pressure equalization in the aorta and disengagement of the catheter during measurement are needed to prevent false negative results. In addition, it is crucial to rule out the presence of any other lesion in the downstream vasculature, as the interplay between the two may impact the assessment on the single lesion [58]. In particular, the presence of a significant lesion distally may reduce blood flow through the LM, thus masking the true gradient across the LM.

After treatment, physiology can be used to assess the result and detect residual ischemia. In case of provisional strategy, FFR can be used to assess the SB and decide whether it needs additional balloon inflation or stent deployment [59]. However, no studies assessed the long-term outcomes of this strategy so far.

## 10. The Risk of Hemodynamic Compromise and Mechanical Support Devices

Given the large myocardial territory it subtends, LM intervention can potentially be complicated by hemodynamic instability, especially in patients with reduced ejection fraction or in those with absent right coronary artery (RCA) support, due to the non-dominance of RCA or chronic total occlusion. Even though most of the patients with LM disease and chronic total occlusion (CTO) of RCA are generally considered candidates for coronary artery bypass grafting (CABG) [60], it is not an infrequent situation to deal with LM PCI in this setting.

Reports show that among patients undergoing LM PCI, patients with concomitant RCA CTO have a worse outcome and a higher mortality rate in comparison to patients without RCA CTO [61,62,63], with RCA CTO being an independent predictor of 3-year cardiac mortality in LM PCI (HR 2.15 [1.02–4.05]; *p* = 0.043) [62]. Furthermore, it was demonstrated that in this population, the recanalization of RCA CTO significantly improves long-term survival [62]. Based on these data, when facing a complex LM PCI, in the case of poor/absent RCA support (RCA stenosis or CTO), an individual approach to each patient is recommended. The use of short-term mechanical support (the so called “protected” PCI) should certainly be considered in the case of complex, diffusely diseased LM PCI with reduced ejection fraction. Furthermore, in patients presenting with a large area of jeopardized myocardium due to significant disease of a dominant, proximal RCA, it is recommended to complete the revascularization, since it may impair late outcome despite successful protected PCI [63,64]. Despite the conflicting results of the use of mechanical circulatory support (MCS) in high-risk PCI, except in the prevention of hemodynamic collapse, short-term MCS (preferably percutaneous devices such as Impella [Abiomed, Danvers, MA, USA], HeartMate PHP [Abbott Vascular Inc., Santa Clara, CA, USA], iVAC2L [PulseCath, Amsterdam; the Netherlands]) should provide adequate time to achieve optimal and reasonably extensive revascularization [65,66].

## 11. Management in Specific Subsets

### 11.1. In-Stent Restenosis

In-stent restenosis (ISR) after ULM-PCI is a frequent finding, and needs specific considerations compared to native disease. Restenosis occurs more often at the ostium of the LCX [67], which is likely due to abnormalities in shear stress, and in the case of complex disease requiring two-stent techniques. First and foremost, it is fundamental to assess the underlying condition of the ISR to better define the treatment strategy. Intracoronary imaging provides essential data and should be considered mandatory. OCT has a slight advantage over IVUS, thanks to its higher spatial resolution.

In particular, ISR can be related to two main conditions: neointima hyperplasia (i.e., proliferation of a new intimal layer inside the stent) and neoatherosclerosis (i.e., the development of a new atherosclerotic plaque, usually with a clear necrotic core, inside the stent). Neointima hyperplasia should be treated with aggressive predilation, often with scoring or cutting balloons, in order to modify the surface of the new intimal layer inside the stent. In case of effective dilation of the lesion (residual stenosis <30%, dissections ≤ grade C, Thrombolysis In Myocardial Infarction [TIMI] flow 3) [68], avoidance of a new stent and prolonged inflation of a drug-coated balloon (DCB) should be considered. DCBs were proven to be non-inferior to drug eluting stent (DES) [31], and have the advantage of avoiding the permanent implantation of a second layer of stent, which may trigger additional stent failure events in the future. On the other hand, in case of ineffective predilation or of neoatherosclerosis, a stent-in-stent implantation may be needed in order to provide sufficient scaffolding to the lesion, as uncoated plain old balloon angioplasty may be burdened by a 2-year rate of target lesion failure up to 40% [68].

### 11.2. Calcified Lesions

Calcified lesions are a frequent finding in the setting of LM disease, and they represent a relevant challenge for the operator, mainly due to the large area of involved myocardium and the risk of severe hypotension when using advanced plaque modification techniques.

In case of need, the operator can use any of the available devices, according to the individual case and vessel anatomy, without any absolute contraindication. Still, it is worth it to highlight that patients with critical ULM disease were excluded from major trials, and only small observational data exist on the topic.

Both rotational and orbital atherectomy are feasible and showed good outcomes after PCI [69,70]. The major risks with these techniques are related to the necessity of removing the safety wire from the side branch, with a consequent higher risk of acute closure of the SB in case of severe disease of both branches or major dissection. Due to the risk of hypotension, atherectomy should always be performed in short runs with adequate intervals to let the heart recover and a limited total length of the procedure to avoid prolonged ischemia or no reflow.

In recent years, intravascular lithotripsy (IVL) emerged as a new technique to address these lesions. The major advantages related to IVL are related to its ease of use, being a balloon-based technique, the low risk of dissections, and the possibility of maintaining the wire in the SB. Even with this device, hypotension may occur during therapy administration, and may require shorter balloon inflations, splitting the treatment cycles (usually 8 cycles of 10 pulses each per catheter) into more cycles composed of fewer pulses. In the limited series, IVL proved to be safe and effective, with a very low rate of adverse procedural and periprocedural events, and good stent expansion [71].

In case of predicted difficulties in treating a calcified lesion with prolonged use of advanced techniques, or persistent hypotension during treatment, the operator should consider short-term circulatory support to maintain adequate values of blood pressure, preserve hemodynamics, and relieve the heart [66].

### 11.3. Acute Coronary Syndromes

Revascularization of the LM, when this is the culprit lesion of an acute coronary syndrome (ACS), is burdened by a higher rate of early mortality and of adverse cardiovascular events, regardless of the modality of treatment. A pooled meta-analysis of the ACS patients from the major available randomized trials comparing PCI and CABG in the setting of LM disease found no differences in 5-year outcomes [72]. The higher risk of adverse events and complications, compared to chronic coronary syndromes (CCS), is mainly due to the large portion of myocardium at potential risk. Phenomena such as no-reflow, dissection, and plaque shift can impair flow in both the LAD and LCX. Sudden deterioration of cardiac function and cardiogenic shock can ensue. Mechanical circulatory support such as Impella or ECMO may be crucial to prevent hemodynamic collapse, and give the operator time to restore coronary flow. In a recent Japanese registry on LM-PCI, cardiogenic shock was the major determinant of cardiac mortality (33.3% at 30 days), while ACS cases without hemodynamic impairment showed only a mild increase (2.5%, compared to 1.1% of CCS) [73]. This high death rate occurred despite a liberal use of mechanical circulatory support devices (intra-aortic balloon pump in 85% of cases, and percutaneous cardiopulmonary support in 26%). Of note, the major procedural difference between groups was no reflow, occurring in 27% of the shock group, significantly higher than ACS without shock (6%) and CCS groups (2.7%). Thus, operators should prevent the development of complications and pursue early resolution in order to avoid potential catastrophic consequences.

## 12. Gaps in Evidence

Despite technical advancements, significant variability in the results of provisional and two-stent strategies exists. In the multitude of available techniques, additional data are needed to guide the operator’s choice in the catheterization laboratory. The definition of a SB prone to occlusion is still variable and elusive, and needs additional studies to better define bifurcations with a low risk of escalation to a second stent and those that would benefit from an upfront, two-stent strategy. Finally, current evidence on the role of physiology guidance is suboptimal. The addition of information from hyperemic/non-hyperemic indexes to guide the choice between treatment and sparing of the SB is intriguing, but needs additional data from large randomized trials.

## 13. Conclusions

Even in the era of modern PCI, treatment of LM lesions remains a challenging scenario, with a consistent rate of adverse events and need for revascularization in the long-term. In-depth knowledge of current indications and proficiency with provisional and upfront two-stent techniques are of critical importance for the achievement of optimal results. Routine adoption of additional tools, especially intracoronary imaging, is strongly suggested to improve procedural results and long-term outcomes.

## Figures and Tables

**Table 1:** Detailed characteristics of current trials comparing surgical and percutaneous revascularization for the treatment of left main disease.

	Boudriot et al. (2003–2009) [23]	SYNTAX (2005–2007) [20,24,25,26,27]	PRECOMBAT (2004–2009) [21,28]	EXCEL (2010–2014) [15,29]	NOBLE (2008–2015) [14,22]
Design	RCT	RCT, subset analysis	RCT, non-inferiority	RCT, non-inferiority	RCT, non-inferiority
Population (patients)	201	700	600	1905	1201
Age (years)	66 (62–73) PCI 69 (63–73) CABG	65.2 ± 9.7 PCI 65.0 ± 9.8 CABG	61.8 ± 10.0 PCI 62.7 ± 9.5 CABG	66.0 ± 9.6 PCI 65.9 ± 9.5 CABG	66.2 ± 9.9 PCI 66.2 ± 9.4 CABG
Male sex	72% PCI 77% CABG	76.4% PCI 78.9% CABG	76.0% PCI 77.0% CABG	76.2% PCI 77.5% CABG	80.0% PCI 76.0% CABG
Diabetes (%)	40% PCI 33% CABG	25.6% PCI 24.6% CABG	34.0% PCI 30.0% CABG	30.2% PCI 28.0% CABG	15% PCI 15% CABG
Previous myocardial infarction (%)	19% PCI 14% CABG	31.9% PCI 33.8% CABG	4.3% PCI 6.7% CABG	18.1% PCI 16.9% CABG	N/A
Left ventricular EF (%)	65.0 (55.0–70.0) PCI 65.0 (55.0–68.0) CABG	N/A (EF < 30% in 1.3% and 2.5 of PCI and CABG group)	61.7 ± 8.3 PCI 60.6 ± 8.5 CABG	57.0 ± 9.6 PCI 57.3 ± 9.0 CABG	60 (55–65) PCI 60 (52–64) CABG
Logistic euroSCORE	2.4 (1.5–3.7) PCI 2.6 (1.7–4.9) CABG	3.8 ± 2.6 PCI 3.8 ± 2.7 CABG	2.6 ± 1.8 PCI 2.8 ± 1.9 CABG	N/A	2 (2–4) PCI 2 (2–4) CABG
SYNTAX Score	24.0 (19.0–29.0) PCI 23.0 (14.8–28.0) CABG	28.4 ± 11.5 PCI 29.1 ± 11.4 CABG	N/A	20.6 ± 6.2 PCI 20.5 ± 6.1 CABG	22.5 ± 7.5 PCI 22.4 ± 8.0 CABG
Use of intracoronary imaging	N/A	4.8%	91.2%	77.2%	74.9%
Trial follow-up	1 year	1 year (extended to 5 years for primary endpoint and 10 years for mortality)	1 year (extended to 10 years)	3 years, (extended to5 years)	5 years
Cutoff for lesion severity	>50% stenosis	>50% stenosis	>50% stenosis	>70% stenosis or FFR ≤0.80	>50% stenosis or FFR ≤0.80
Composite Primary endpoint	Death MI Repeat revascularization	Death Stroke MI Repeat revascularization	Death Stroke MI Repeat revascularization	Death Stroke MI	Death Stroke Non-procedural MI Repeat revascularization
Results	19.0% PCI vs. 13.9% CABG	5-year primary endpoint: 15.8% PCI vs. 13.7% CABG 10-year mortality 27% vs. 28%	29.8% PCI vs. 24.7% CABG	22.0% PCI vs. 19.2% CABG	29% PCI vs. 19% CABG, *p* 0.007. HR 1.48 (95% CI 1.11–1.96)

CABG: coronary artery bypass graft. EF: ejection fraction. FFR: fractional flow reserve. HR: hazard ratio. MI: myocardial infarction. PCI: percutaneous coronary intervention. RCT: randomized controlled trial.

## Data Availability

Not applicable.
